# Association between changes in hip-knee-ankle angle and hindfoot alignment after total knee arthroplasty for varus knee osteoarthritis

**DOI:** 10.1186/s12891-021-04488-y

**Published:** 2021-07-06

**Authors:** Naicheng Diao, Fei Yu, Bo Yang, Lifeng Ma, Heyong Yin, Ai Guo

**Affiliations:** Department of Orthopaedics, Beijing Friendship Hospital, Capital Medical University, No. 95, Yongan Road, Xicheng District, 100050 Beijing, China

**Keywords:** Total knee arthroplasty, Hindfoot alignment, Hip-knee-ankle angle, Varus deformity, Osteoarthritis

## Abstract

**Background:**

The change in hip-knee-ankle (HKA) angle after total knee arthroplasty (TKA) may cause an adjustment in hindfoot alignment (HFA). However, the relationship between the changes in HKA angle and HFA is still not well studied. This study aimed to investigate the association between HKA angle and hindfoot alignment changes after TKA for varus knee osteoarthritis.

**Methods:**

A prospective study was carried out in which 108 patients with varus knee deformities were radiographically and clinically evaluated before and 3 months after TKA. The relationship of change in HFA with correction in HKA angle was investigated.

**Results:**

The results showed that the HFA was adjusted significantly by 3 months after TKA (*p* < 0.001), along with improved American Orthopaedic Foot and Ankle Society (AOFAS) ankle hindfoot score (*p* < 0.001). Next, a univariate correlation and linear regression analysis showed that the change in HFA was weakly correlated with the change in HKA angle (*r*=-0.262, β=-0.14, 95 % CI: -0.23 to -0.04, *P* = 0.006). Further stratified analysis and interaction tests revealed that age has a distinct effect on the correlation between the changes in HFA and HKA angle. The correlation was dramatically greater in the group under 65 years (*r*=-0.474, β=-0.26, 95 % CI: -0.41 to -0.12, *P* = 0.001), whilst, no correlation was observed in those above 65 years old (*r*=-0.036, β=-0.02, 95 % CI: -0.14 to 0.11, *P* = 0.779).

**Conclusions:**

Our findings indicated that correction of HKA after TKA tend to promote adjustment in the hindfoot alignment toward re-balance of the whole lower limb weight-bearing axis. However, this mechanism obviously weakens in elderly patients. Therefore, if apparent hindfoot deformity exists in these patients before TKA, more perioperative intervention is required for hindfoot adjustment, and even HKA undercorrection may be considered.

## Background

TKA is the most common surgical procedure for the end-stage osteoarthritic knee [1–3]. The HKA angle representing the mechanical alignment that runs from the femoral head center to ankle center is expected to be adjusted toward neutral after TKA [4]. However, the weight-bearing axis of the lower extremity that goes from pelvis to the ground including the hindfoot is drawing more and more attention, because it also determines the improvement of the clinical symptoms and long-term survival of the implant [5]. The total lower extremity weight-bearing axis is affected by the hindfoot alignment, and the hindfoot varus or valgus deformities lead to change of overall lower extremity weight-bearing axis [6–8], which may cause secondary clinical problems. This emphasizes the importance of hindfoot alignment for the whole lower extremity loading axis.

Varus deformity of the knee is prevalent in patients with severe osteoarthritis. To date, several studies have reported that patients with knee varus deformity develop compensatory changes at the subtalar joints by changing the joint toward valgus [9–11]. Okamoto et al [12] reported that patients with severe knee deformity usually underwent persistent foot pain after TKA if the hindfoot alignment was not well corrected. Several studies showed that knee deformities are closely connected with feet deformities, and TKA affected the axis of the lower limb with adjustment in the hindfoot alignment after operation [11, 13, 14]. So far, the detailed mechanism and association of HKA angle and hindfoot alignment post-operation remain controversial. A clear understanding of the association between the adjustments in the hindfoot alignment with the change in lower extremity mechanical alignment is critically essential to predict and prevent negative impacts on the overall lower extremity weight-bearing axis.

The current study hypothesized that correction of HKA angle after TKA affects hindfoot alignment toward neutral mechanical alignment of the whole lower extremity. Therefore, this study aims to investigate the association between the changes in HKA angle and hindfoot alignment after TKA. Besides, variations of function and clinical symptoms in ankle and hindfoot would be evaluated by American Orthopaedic Foot and Ankle Society (AOFAS) ankle hindfoot score. Since the hindfoot was getting more rigid with ageing, it would make sense to investigate if the elderly patients lose the compensatory capacity. And hardly any studies have focused on the impact of age on these changes.

Based on above, the main objectives of this study were to determine: (1) the changes of HKA angle and HFA after TKA; (2) the association between the changes in HKA angle and HFA; (3) the impact of age on these changes.

## Methods

### Patient enrollment

**Fig. 1:**
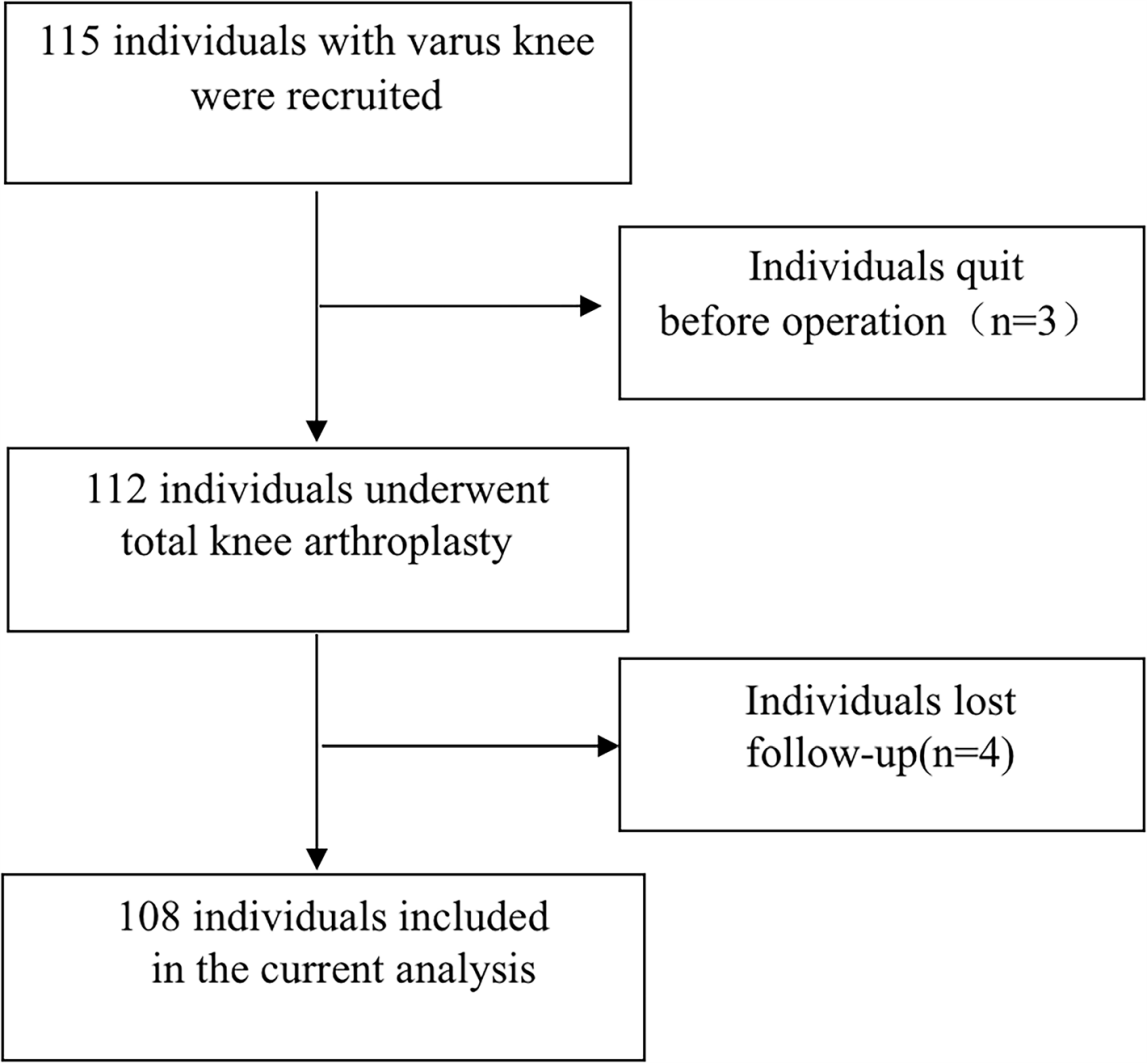
Flowchart of the participants in this study

This prospective study was approved by, and conducted in strict accordance with the regulations of our institutional Research Ethics Committee. Written consent was obtained from all the enrolled individuals. The flow chart of the participants is presented in Fig. 1. Totally, 108 eligible participants (aged from 50 to 80 years old) who underwent TKA for varus knee osteoarthritis from July 2017 to June 2018 were enrolled. There were 12 patients complained with foot symptoms before TKA. Each participant underwent unilateral TKA by one skilled orthopaedic surgeon. Briefly, posterior-stabilized (PS) TKA using an intramedullary guide for distal femoral cut, and extramedullary guide for tibial cut were performed to achieve the surgical goals of correcting the HKA angle towards 0°. Patients who were diagnosed with rheumatoid arthritis, post-traumatic arthritis, congenital deformities in the limbs, or those who underwent previous ankle or hindfoot surgery were excluded for this study. Patients with rigid foot were also excluded after physical examination by two experienced foot and ankle surgeons.

The patient information, including age, sex, height, weight, body mass index (BMI), was collected. In addition, radiological evaluation before and three months after operation was conducted for all the enrolled patients, and the clinically relevant AOFAS Ankle Hindfoot Scale was performed before and 3 months after TKA as well. Radiographic images were digitized and stored in the Electronic Medical Record System. Image measurement software (UniWeb Viewer, EBM Technologies, Inc.) was used for the measurements of the HKA angle and hindfoot alignment. Individually, preoperative HKA angle (HKAB) and postoperative HKA (HKAP), change in HKA angle (CHKA = HKAP- HKAB), preoperative HFA (HFAB) and postoperative HFA (HFAP), as well as the change in HFA (CHFA = HFAP- HFAB) were measured. The AOFAS score involves nine different items regarding pain, function and mobilization, and joint alignment with a maximum score of 100 points. A higher score represents a better clinical performance of the ankle and hindfoot.

### Radiological evaluation

**Fig. 2:**
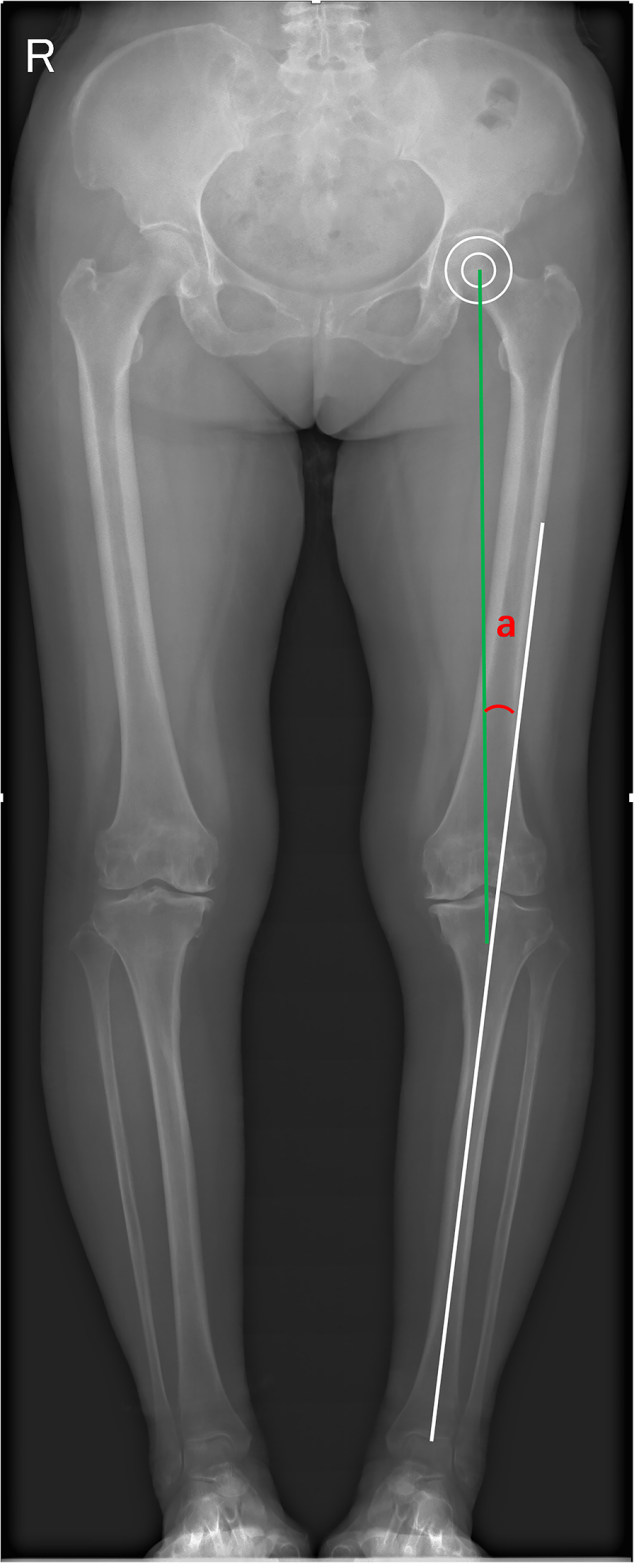
HKA angle measurement on a full-leg standing anteroposterior (AP) radiograph. HKA angle(a) was defined as the angle formed by the intersection of a line drawn from the center of the femoral head to the intercondylar fossa of the distal femur (green line) and a line drawn from the intercondylar spine of the proximal tibia to the center of the ankle joint (white line)

**Fig. 3:**
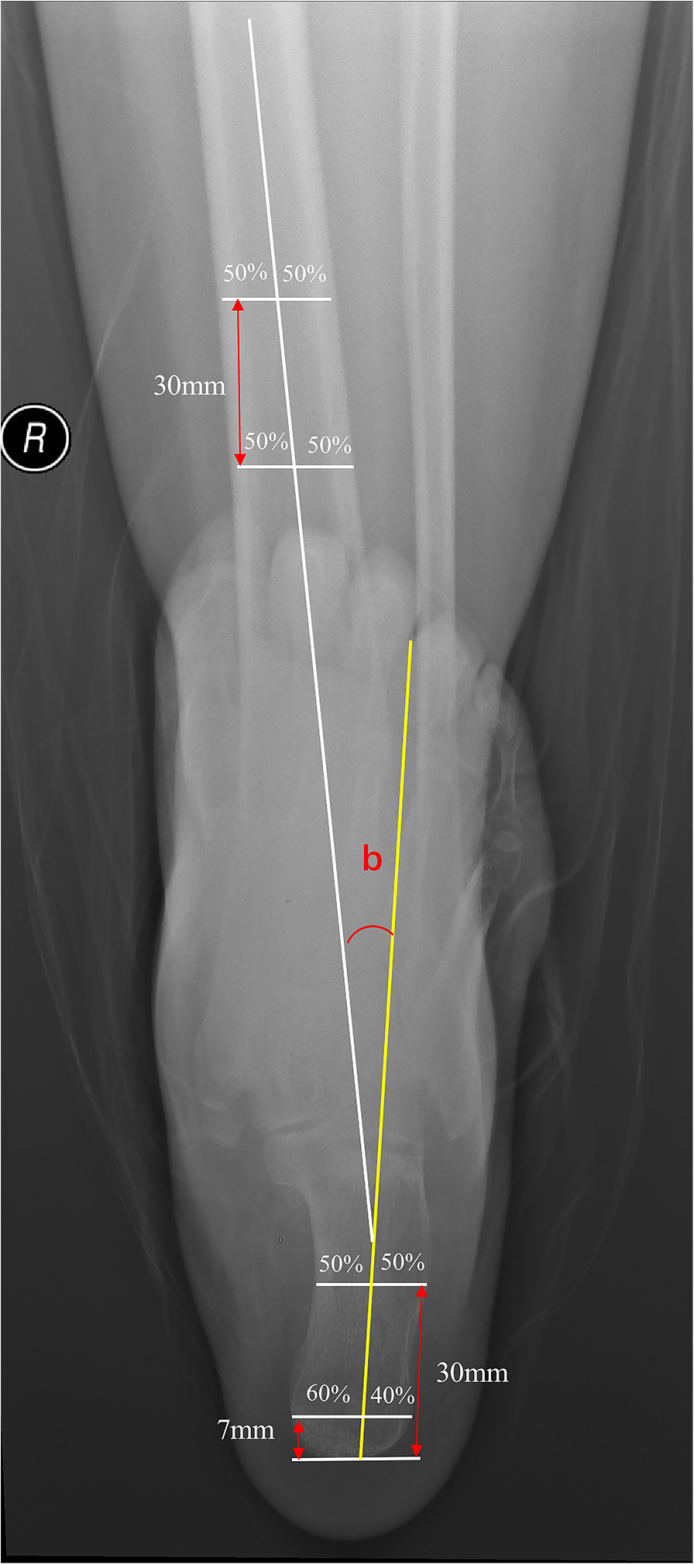
Hindfoot alignment measurement on a long axial view of the hindfoot. Hindfoot alignment(b) was measured as the angle between the anatomical axis of the tibia (long white line) and the axis of the calcaneus (yellow line). The tibia mid-diaphyseal axis was determined by the extending line through two mid-diaphyseal points (30 mm apart) that bisect the tibia. The calcaneal axis was presented by the extending line of two points: one of the points is the 40 % mark of a horizontal line7 mm from the most distal part of the calcaneus (divided into 40 %:60 % ratio), the other point is the bisecting point of a horizontal line 30 mm from the most distal part of the calcaneus

The hindfoot alignment and HKA angle were assigned as a negative value for varus and a positive value for valgus. HKA angle was measured from full-leg length standing anteroposterior (AP) radiographs. HKA indicating the alignment of the lower limbs was defined as the angle between the femur mechanical axis, a line drawn from femoral head center to center of the knee and tibia mechanical axis, a line drawn from center of the knee to center of ankle joint (Fig. 2) [13, 15]. The weight-bearing hindfoot alignment radiographs were obtained from the long axial view. This method has been widely used in many studies because it demonstrates hindfoot alignment well [16–18]. The hindfoot alignment was measured as the intersectant angle of two lines: one line indicates the tibia mid-diaphyseal axis and the other line indicates the mid-diaphyseal calcaneus axis. The tibia mid-diaphyseal axis was determined by the extending line through two mid-diaphyseal points (30 mm apart) that bisect the tibia. The calcaneal axis was presented by the extending line of two points: one of the points is the 40 % mark of a horizontal line7 mm from the most distal part of the calcaneus (divided into 40 %:60 % ratio), the other point is the bisecting point of a horizontal line 30 mm from the most distal part of the calcaneus (Fig. 3).

### Statistical analysis

Continuous variables were expressed as median or mean ± standard deviation (SD) and categorical variables were presented as frequency (%). Student’s t-test was used to compare the HKA angle, hindfoot alignment, and AOFAS score before and three months after TKA. A univariate linear regression model was applied to evaluate the association between the change in HKA angle and the change in HFA. Furthermore, stratified analysis and interaction tests were conducted for age, sex, BMI, preoperative HKA, postoperative HFA to determine the potential factors that affect the association between the change in HKA and change in HFA. The WHO has defined “elderly” as a chronological age of 65 years old or older. The BMI score is used to determine whether a person has too much body fat. BMI scores of 20 to 24.9 are considered normal or healthy weight. scores over 25 are overweight. Thus, to determine whether age and weight have an impact on the correlation between the change in HKA angle and hindfoot alignment. We divided a further stratified analysis and interaction tests into age of < 65 years and ≥ 65 years, BMI of < 25 kg/m2 and ≥ 25 kg/m2.In addition, refer to literature [10], there was a moderate negative association between the HKA angle and the Saltzman hindfoot angle, but this trend was only limited for patients with severe knee deformities (more than 10°). And hindfoot alignment HFA > 0° indicates valgus hindfoot, while HFA < 0° indicates varus hindfoot. Therefore, preoperative HKA of ≤-10° and >-10°, or preoperative HFA of ≤ 0° and > 0° were also determined for stratified analysis and interaction tests. The statistical analysis was performed using the software packages R version 3.4.3 (http://www.R-project.org, The R Foundation) and Empower States (http://www.empowerstates.com, X & Y Solutions, Inc., Boston, MA). *P* values less than 0.05 (two-sided) were considered statistically significant. The required sample size was determined by a power calculation using PASS 15.0. The calculation was conducted based on the Pearson correlation, *r* = − 0.347, which was reported by Norton et al [10] and includes the following assumption: alpha of 0.05 (two-tailed) and power of 0.9 revealing a minimum of 83 patients required to detect a difference in correlation between HKA angle and hindfoot alignment changes in varus knee after TKA.

An intra- and inter-observer variability assessment was conducted to validate the methodology regarding the radiographic measurements by using intraclass correlation coefficients. Totally 20 (18.5 %) subjects were selected from the overall study cohort (*n* = 108) for remeasurement. The intra- and inter-observer coefficients for HKA angle and HFA were 0.918 and 0.915, and 0.986 and 0.935, respectively.

## Results

### Demographics, description of study population

Among the 108 patients, there were 89 females and 19 males. The mean age of the participants at the time of TKA was 66.31 ± 5.55 years (range 56–79 years). The mean BMI was 27.88 ± 3.65 (range 20.21–40.01). The mean changes in HKA angle and hindfoot alignment were 6.77 ± 5.15° and − 2.16 ± 2.70°, respectively.

### The HKA angle and hindfoot alignment changed significantly after TKA with varus knee

HKA angle was significantly improved from − 9.15° ±5.54 to -2.37° ± 3.05 by three months post-operation (*p* < 0.001). Interestingly, the hindfoot alignment was significantly adjusted from 2.67°± 3.97 to 0.52°± 3.96 (*p* < 0.001). Moreover, TKA showed a significant positive effect on the AOFAS score from 76.50 ± 10.89 before the operation to 90.40 ± 6.05 post-operation (*p* < 0.001) (Table 1).

**Table 1:** Pre- and post-operative HKA angle, hindfoot alignment and AOFAS score

Variables	Pre-operative	Post-operative	*P*-value
HKA	-9.15 ± 5.54	-2.37 ± 3.05	<0.001*
HFA	2.67 ± 3.97	0.52 ± 3.96	<0.001*
AOFAS	76.50± 10.89	90.40± 6.05	<0.001*

Abbreviations: *HKA* Hip-Knee-Ankle, *HFA* hindfoot alignment, *AOFAS* The American Orthopaedic Foot & Ankle Society

*indicates statistical significance at *P* <0.05

### The change in hindfoot alignment was correlated with the change in HKA angle after TKA with varus knee

A univariate linear regression analysis revealed that the change in HFA was significantly correlated with change in HKA (*P* = 0.006). However, the correlation was very weak (*r*= -0.262). It indicated that the correction of HKA angle from varus to neutral by operation led to subsequent compensation of the hindfoot alignment by adjusting from valgus to neutral, with every degree changed in HKA angle followed by -0.14° adjustment in the hindfoot alignment (β= -0.14, 95 % CI: -0.23 to -0.04) (Table 2).

**Table 2:** Global correlation between CHKA and CHFA

Variables	r	*P-*value	β(95%CI)	*P-*value
CHFA-CHKA	-0.262	0.006*	-0.14 (-0.23, -0.04)	0.006*

Abbreviations: *CHKA* change in Hip-Knee-Ankle angle, *CHFA* change in hindfoot alignment

*indicates statistical significance at *P* <0.05

### Age showed a distinct impact on the correlation of changes in HKA and HFA after TKA with varus knee

**Fig. 4:**
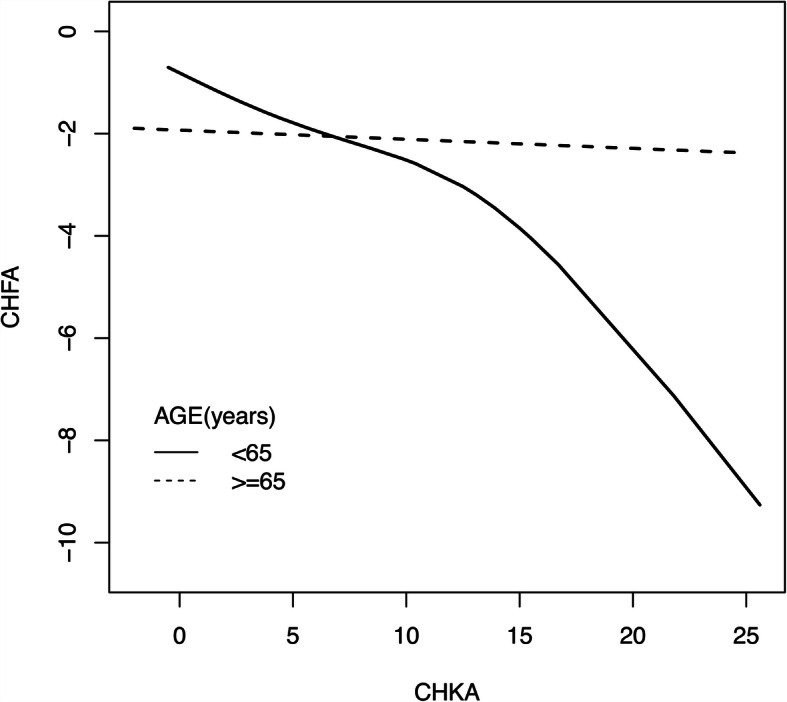
Age affected the association between the change in HKA (CHKA) and change in HFA (CHFA). The smooth curves indicated a correlation between the CHKA and CHFA in the group under 65 years. However, no correlation was detected in the group older than 65 years. Abbreviations: CHKA, change in Hip-Knee-Ankle angle; CHFA, change in hindfoot alignment.

To determine the potential factors that affect the correlation between the change in HKA and change in HFA, a further stratified analysis and interaction tests were performed by age (< 65, ≥ 65 years), gender (female, male), BMI (< 25, ≥ 25 kg/m^2^), preoperative HKA (≤-10^0^, >-10^0^) and preoperative HFA (≤ 0^0^, >0^0^). The results indicated that age has a distinct impact on the correlation between the change in HKA and change in HFA (P for interaction = 0.001). Besides, the correlation was dramatically higher in the group under 65 years (*r*= -0.474), with every degree changed in HKA angle followed by -0.26° adjustment in the hindfoot alignment (β= -0.26, 95 % CI: -0.41 to -0.12, *P* = 0.001). In those patients above 65 years old, there was no clear correlation between the change in HKA and change in HFA (*r*=-0.036, β=-0.02, 95 % CI: -0.14 to 0.11, *P* = 0.779) (Table 3). The smooth curve also showed that the distinct correlation between the changes in HKA and hindfoot alignment was only observed in the patients younger than 65 years old (Fig. 4). The stratified analysis showed no significant impacts of sex, BMI, preoperative HKA, and preoperative HFA on the correlation of changes in HKA and hindfoot alignment (*P* for interaction > 0.05) (Table 3).

**Table 3:** Association between CHKA and CHFA according to baseline characteristics

Characteristic	n	r	β(95%CI)	*P-*value	*P* for interaction
Age(year)					0.010*
<65	46(42.59%)	-0.474	-0.26 (-0.41, -0.12)	0.001	
≥65	62(57.41%)	-0.036	-0.02 (-0.14, 0.11)	0.779	
Sex					0.753
female	89(82.41%)	-0.247	-0.13 (-0.24, -0.02)	0.020	
male	19(17.59%)	-0.316	-0.19 (-0.46, 0.08)	0.187	
BMI (kg/m2)					0.101
<25	24(22.22%)	-0.05	-0.02 (-0.17, 0.14)	0.824	
≥25	84(77.78%)	-0.32	-0.19 (-0.31, -0.07)	0.002	
HKAB (^0^)					0.237
≤ -10	40(37.04%)	-0.308	-0.15(-0.30, -0.00)	0.054	
> -10	68(62.96%)	0.001	0.00 (-0.21, 0.21)	0.992	
HFAB (^0^)					0.527
≤0	26(24.07%)	-0.054	-0.11 (-0.28, 0.06)	0.205	
>0	82(75.93%)	-0.298	-0.18(-0.28, -0.07)	0.003	

Abbreviations: *CHKA* change in Hip-Knee-Ankle angle, *CHFA* change in hindfoot alignment, *BMI* body mass index, *HKAB* preoperative Hip-Knee-Ankle angle, *HFAB* preoperative hindfoot alignment

*indicates statistical significance at *P* <0.05

## Discussion

The most important finding of the current study was that hindfoot alignment tend to adjust with improved AOFAS ankle hindfoot score after the correction of HKA, as a result of a residual compensatory capacity in the hindfoot. However, the compensatory capacity obviously weakens in elderly patients.

There is no consensus yet about the appropriate hindfoot alignment after TKA. Since the HKA angle representing the mechanical alignment that runs from the femoral head center to ankle center is expected to be adjusted toward neutral after TKA. And the weight-bearing axis of the lower extremity that goes from pelvis to the ground including the hindfoot also determines the improvement of the clinical symptoms and long-term survival of the implant. It is expected that the hindfoot alignment would adjust towards 0°, as a result of a residual compensatory capacity in the hindfoot and thus to achieve a neutral mechanical alignment of the whole lower extremity. Norton et al [10] reported that when the HKA angle becomes varus, the hindfoot tend to shift toward valgus subsequently. Hara et al [19] divided 100 patients with varus osteoarthritis knee into two groups before operation according to the hindfoot alignment deformities (varus or valgus). They found that the hindfoot alignment improved significantly after TKA in the hindfoot valgus group, but not in the varus group. The current study indicated similar findings as well. Firstly, our results showed that most of the patients with osteoarthritis varus knees accompanied with valgus hindfoot (75.93 %). Furthermore, compensative change was also found to occur in hindfoot with significant adjustment in the hindfoot alignment from 2.67°± 3.97 to 0.52°± 3.96 (*p* < 0.001) by three months after TKA. This finding is quite important since the hindfoot alignment is not included for the traditional mechanical axis of the lower limb. An abnormal hindfoot alignment will negatively affect the overall weight-bearing axis which goes from pelvis to the ground, causing secondary clinical complaints and affecting long-term survival of implants, even though satisfactory neutral mechanical alignment is achieved by the surgery [12, 20]. Besides the change in hindfoot alignment, the current study also evaluated the alterations of clinical symptoms and function in the ankle hindfoot by AOFAS score. These results indicated that a significant improvement in AOFAS score was achieved along with the adjustment of hindfoot alignment after TKA (*P* < 0.001).

Based on above, it is critically important to further investigate the clear associations between the changes in HKA angles and hindfoot alignment after TKA. Chandler et al [21] reviewed 100 cases by three months after TKA. They found that even though the hindfoot alignment was adjusted after TKA, the change in hindfoot alignment did not correlate significantly with the change in HKA angle. However, Norton et al [10] reported that there was a moderate negative association between the HKA angle and the Saltzman hindfoot angle, but this trend was only limited for patients with severe knee deformities (more than 10°). In the current study, a univariate linear regression analysis demonstrated that the change in hindfoot alignment was weakly associated with the change in HKA angle after TKA with varus knee (*r*=-0.262, β=-0.14, 95 % CI: -0.23 to -0.04, *P* = 0.006). In addition, our results also indicated that correlation between the changes in HKA angle and hindfoot alignment was higher for those with more severe knee deformities (more than 10°), however no statistical significance was detected (*p* > 0.05).

Since the hindfoot becomes more rigid with ageing, which may weaken the compensatory capacity in the elderly patients, our study further investigated whether age will affect the correlation of the change in hindfoot alignment and change in HKA angle after TKA. As expected, a stratified analysis showed that age has a distinct effect on this association (*P* for interaction = 0.010). The change in the hindfoot alignment was moderately correlated with the change in HKA angle for patients below 65 years old (*r*=-0.474, β=-0.26, 95 % CI: -0.41 to -0.12, *P* = 0.001). However, for patients over 65 years old, no correlation between these changes was found (*r*=-0.036, β=-0.02, 95 % CI: -0.14 to 0.11, *P* = 0.779), which confirmed that the compensative capacity weakens in elderly patients. As far as we know, this is the first study that revealed the effect of age on the association between the changes in the hindfoot alignment and HKA angle after TKA. Therefore, evaluation of hindfoot alignment and its relationship to the knee deformity should be taken into consideration prior to TKA for patients with varus knee osteoarthritis. For the patients under 65 years old, additional surgery for correcting the hindfoot alignment may not be recommended, whilst, for those over 65 years old, more attention for hindfoot alignment is required. More specifically, if obvious hindfoot valgus deformity exists in the patient elder than 65 years, the deformity tends to persist after TKA, which will then affect the whole lower limb weight-bearing axis. The abnormal lower limb weight-bearing axis may lead to secondary clinical complaints and affect the survival of implants. To avoid this problem, extra treatments, such as insoles or orthotics are recommended for these patients to obtain a better balance of the lower limb axis. Moreover, undercorrection could be considered during TKA in varus osteoarthritis for the elderly, especially for those with rigid hindfeet in the physical examination.

Nevertheless, some limitations of the study need to be addressed. First, the follow-up of three months is a relatively short period, since the hindfoot alignment and the clinical symptoms can be changed with time. However, refer to literature, the time point of three months is adequate to draw a definite conclusion in the study for HKA angle and hindfoot alignment changes after TKA [9, 11]. Second, the exact mechanism of how hindfoot alignment change will affect the overall loading axis of the lower limb is not explored. Third, the criteria of rigid hindfoot were assessed only through physical examination by two experienced foot and ankle surgeons. Whilst, possible bone deformities, especially in the subtalar joint, were not adequately evaluated. Moreover, the AOFAS score may not reflect the alteration of hindfoot function so accurately since the score including pain and walking distance items which may be influenced by pain in the knee. Lastly, it is hard to distinguish the exact anatomic location of calcaneus from the full-leg length standing anteroposterior radiographs in the current study, for which the measurement of the HKA angle and hindfoot alignment were performed using two different tibial axes. In the future work, we would try to locate the calcaneus by some additional labels during the radiographing of full-leg length standing anteroposterior view. It would make it possible to provide the same tibial axe for the measurement of HKA angle and hindfoot alignment.

## Data Availability

The datasets used and analyzed during the current study are available from the corresponding author on reasonable request.

## Abbreviations

HKA: Hip-knee-ankle
TKA: Total knee arthroplasty
HFA: Hindfoot alignment
AOFAS: American Orthopaedic Foot and Ankle Society
BMI: Body mass index
HKAB: Preoperative Hip-Knee-Ankle angle
HKAP: Postoperative Hip-Knee-Ankle angle
HFAB: Preoperative hindfoot alignment
HFAP: Postoperative hindfoot alignment
CHKA: Change in Hip-Knee-Ankle angle
CHFA: Change in hindfoot alignment
AP: Anteroposterior

## References

[1] Jain S, Pathak AC, Kalaivanan K (2016). Minimum 5-year follow-up results and functional outcome of rotating-platform high-flexion total knee arthroplasty: A prospective study of 701 knees. Arthroplast Today.

[2] Solarino G, Spinarelli A, Carrozzo M, Piazzolla A, Vicenti G, Moretti B (2014). Long-term outcome of low contact stress total knee arthroplasty with different mobile bearing designs. Joints.

[3] Zhang Z, Liu C, Li Z, Wu P, Hu S, Liao W. Residual Mild Varus Alignment and Neutral Mechanical Alignment Have Similar Outcome after Total Knee Arthroplasty for Varus Osteoarthritis in Five-Year Follow-Up. J Knee Surg. 2020;33(2):200–5. 10.1055/s-0038-1677497 30650442

[4] Almaawi AM, Hutt JRB, Masse V, Lavigne M, Vendittoli PA (2017). The Impact of Mechanical and Restricted Kinematic Alignment on Knee Anatomy in Total Knee Arthroplasty. J Arthroplasty..

[5] Meding JB, Keating EM, Ritter MA, Faris PM, Berend ME, Malinzak RA (2005). The planovalgus foot: a harbinger of failure of posterior cruciate-retaining total knee replacement. J Bone Joint Surg Am.

[6] Desai SS, Shetty GM, Song HR, Lee SH, Kim TY, Hur CY (2007). Effect of foot deformity on conventional mechanical axis deviation and ground mechanical axis deviation during single leg stance and two leg stance in genu varum. Knee..

[7] Guichet JM, Javed A, Russell J, Saleh M. Effect of the foot on the mechanical alignment of the lower limbs. Clin Orthop Relat Res. 2003;415(415):193–201. 10.1097/01.blo.0000092973.12414.ec 14612646

[8] Lee ST, Song HR, Mahajan R, Makwana V, Suh SW, Lee SH (2007). Development of genu varum in achondroplasia: relation to fibular overgrowth. J Bone Joint Surg Br.

[9] Cho WS, Cho HS, Byun SE (2017). Changes in hindfoot alignment after total knee arthroplasty in knee osteoarthritic patients with varus deformity. Knee Surg Sports Traumatol Arthrosc.

[10] Norton AA, Callaghan JJ, Amendola A, Phisitkul P, Wongsak S, Liu SS, Fruehling-Wall C (2015). Correlation of knee and hindfoot deformities in advanced knee OA: compensatory hindfoot alignment and where it occurs. Clin Orthop Relat Res..

[11] Takenaka T, Ikoma K, Ohashi S, Arai Y, Hara Y, Ueshima K, Sawada K, Shirai T, Fujiwara H, Kubo T (2016). Hindfoot alignment at one year after total knee arthroplasty. Knee Surg Sports Traumatol Arthrosc..

[12] Okamoto Y, Otsuki S, Jotoku T, Nakajima M, Neo M (2017). Clinical usefulness of hindfoot assessment for total knee arthroplasty: persistent post-operative hindfoot pain and alignment in pre-existing severe knee deformity. Knee Surg Sports Traumatol Arthrosc.

[13] Jeong BO, Kim TY, Baek JH, Jung H, Song SH (2018). Following the correction of varus deformity of the knee through total knee arthroplasty, significant compensatory changes occur not only at the ankle and subtalar joint, but also at the foot. Knee Surg Sports Traumatol Arthrosc.

[14] Mansur H, Rocha FA, Garcia PBL, de Alencar FHU, Guilme P, de Castro IM. Alteration of Hindfoot Axis After Total Knee Arthroplasty. J Arthroplasty. 2019;34(10):2376–82. 10.1016/j.arth.2019.05.029 31262620

[15] Moreland JR, Bassett LW, Hanker GJ (1987). Radiographic analysis of the axial alignment of the lower extremity. J Bone Joint Surg Am.

[16] Lamm BM, Mendicino RW, Catanzariti AR, Hillstrom HJ (2005). Static rearfoot alignment: a comparison of clinical and radiographic measures. J Am Podiatr Med Assoc.

[17] Neri T, Barthelemy R, Tourne Y (2017). Radiologic analysis of hindfoot alignment: Comparison of Meary, long axial, and hindfoot alignment views. Orthop Traumatol Surg Res.

[18] Reilingh ML, Beimers L, Tuijthof GJ, Stufkens SA, Maas M, van Dijk CN (2010). Measuring hindfoot alignment radiographically: the long axial view is more reliable than the hindfoot alignment view. Skeletal Radiol..

[19] Hara Y, Ikoma K, Arai Y, Ohashi S, Maki M, Kubo T (2015). Alteration of hindfoot alignment after total knee arthroplasty using a novel hindfoot alignment view. J Arthroplasty..

[20] Mullaji A, Shetty GM (2011). Persistent hindfoot valgus causes lateral deviation of weightbearing axis after total knee arthroplasty. Clin Orthop Relat Res..

[21] Chandler JT, Moskal JT (2004). Evaluation of knee and hindfoot alignment before and after total knee arthroplasty: a prospective analysis. J Arthroplasty..

